# Cardamonin reduces chemotherapy-enriched breast cancer stem-like cells *in vitro* and *in vivo*

**DOI:** 10.18632/oncotarget.5819

**Published:** 2015-10-17

**Authors:** Deyong Jia, Yuan Tan, Huijuan Liu, Sarah Ooi, Li Li, Kathryn Wright, Steffany Bennett, Christina L. Addison, Lisheng Wang

**Affiliations:** ^1^ Department of Biochemistry, Microbiology and Immunology, Faculty of Medicine, University of Ottawa, Ottawa, Ontario K1H 8M5, Canada; ^2^ Bio-X Institutes, Key Laboratory for the Genetics of Developmental and Neuropsychiatric Disorders, Ministry of Education, Shanghai Jiao Tong University, Shanghai 200240, China; ^3^ Centre for Cancer Therapeutics, Ottawa Hospital Research Institute, Ottawa, Ontario K1H 8L6, Canada; ^4^ Regenerative Medicine Program, Ottawa Hospital Research Institute, Ottawa, Ontario K1H 8L6, Canada; ^5^ Ottawa Institute of Systems Biology, University of Ottawa, Ottawa, Ontario K1H 8M5, Canada

**Keywords:** chemotherapy, cardamonin, breast cancer stem cells, in vivo

## Abstract

The failure of cytotoxic chemotherapy in breast cancers has been closely associated with the presence of drug resistant cancer stem cells (CSCs). Thus, screening for small molecules that selectively inhibit growth of CSCs may offer great promise for cancer control, particularly in combination with chemotherapy. In this report, we provide the first demonstration that cardamonin, a small molecule, selectively inhibits breast CSCs that have been enriched by chemotherapeutic drugs. In addition, cardamonin also sufficiently prevents the enrichment of CSCs when simultaneously used with chemotherapeutic drugs. Specifically, cardamonin effectively abolishes chemotherapeutic drug-induced up-regulation of IL-6, IL-8 and MCP-1 and activation of NF-κB/IKBα and Stat3. Furthermore, in a xenograft mouse model, co-administration of cardamonin and the chemotherapeutic drug doxorubicin significantly retards tumor growth and simultaneously decreases CSC pools *in vivo*. Since cardamonin has been found in some herbs, this work suggests a potential new approach for the effective treatment of breast CSCs by administration of cardamonin either concurrent with or after chemotherapeutic drugs.

## INTRODUCTION

Breast cancer is the most prevalent cancer diagnosed among women in the world. In the United States, approximately more than 40,000 women will die of breast cancer in 2015 [1]. Chemotherapeutics are a standard of care in clinical oncology today due to their effectiveness in reducing tumor burden and improving survival [2]. However, the disease often relapses, with only a 23% 5-year survival rate in these patients with breast cancer [3–5].

Accumulating evidence suggests that a subset of tumor cells capable of initiating new tumors, called cancer stem cells (CSCs), are a major barrier for tumor control in patients refractory to drug treatment [6–9]. However, current treatments mainly target the bulk of the tumor cells rather than CSCs, which may lead to drug resistance and subsequent recurrence and metastasis [10]. Several recent studies have shown that breast CSCs could be enriched following chemotherapy [5, 11–13], suggesting that complete eradication of CSCs is necessary to achieve a cure [6–9]. As such, a new strategy combining conventional tumor-shrinking cytotoxic drugs and CSC-targeting agents may lead to better disease control and prevent breast cancer relapse.

Recently, the use of natural phytochemicals to impede tumors has gained immense importance [14]. Cardamonin, found in cardamom spice and many other plant species, has been studied extensively as a chemo-preventive agent in a variety of cancers, including breast, hematological, gastrointestinal, and colorectal cancers [15]. However, the association between CSCs and cardamonin has not been reported. We recently screened large numbers of small molecules and found that cardamonin could modestly inhibit tumor growth while effectively abrogating the breast CSC population that had been enhanced after poly(I:C) treatment of breast cancer cells *in vitro* and *in vivo* [16]. We further observed that cardamonin could significantly inhibit the expression of stem-marker genes in breast cancer cells. We therefore postulated that cardamonin might selectively repress the enrichment of breast CSCs induced by drug treatment and enhance the efficacy of chemotherapeutic treatments.

In this report, we show that treatment with first-line chemotherapeutic drugs markedly enriches the breast CSC subpopulation. We also demonstrate that cardamonin selectively reduces the population of CSCs enriched by first-line chemotherapeutic drugs in different types of breast cancer cells. Furthermore, simultaneous use of cardamonin and chemotherapeutic drugs also prevents the generation of new CSCs. Cardamonin abolishes the phosphorylation of NF-κB/IKBα and Stat3 and represses the up-regulation of IL-6, IL-8 and MCP-1 cytokines induced by drug treatments. Importantly, co-administration of cardamonin and the chemotherapeutic drug doxorubicin markedly retards tumor growth while inhibiting CSC pools in a xenograft mouse model. These findings provide a rationale and experimental basis for the combinational use of tumor-shrinking drugs and CSC-targeting agents in clinical settings.

## RESULTS

### Chemotherapeutic drugs enrich breast CSC subpopulation

Among chemotherapeutic drugs used widely for patients with breast cancers, 5-fluorouracil (targeting thymidylate synthase and DNA synthesis), doxorubicin (targeting topoisomerase II), and paclitaxel (targeting cytoskeleton structure tubulin) are three well-defined first-line agents [17, 18]. To test whether these first-line chemotherapeutic drugs enrich breast CSCs, we treated breast cancer cells with three individual drugs for 4d followed by a 2d recovery, and then conducted subsequent assays to determine presence and characteristics of CSCs in the population in the absence of additional treatment according to previously established procedures [7]. Drug concentrations used in this study were chosen based on three factors: clinically relevant concentrations [13, 19–22], titration determining approximately half maximal inhibitory concentrations using SUM190, MCF-7, and Cama-1 breast cancer cell lines (Supplementary Figure S1), and the potential to enrich breast CSCs.

We analyzed CSC properties by flow cytometry, quantitative real-time PCR (Q-PCR), Western blot, and mammosphere formation. Since a single marker is not sufficient for characterization of CSC due to high inter-patient and intra-tumor variability, we employed various markers which have been used regularly in the field, such as CD44^high^/CD24^−/low^ expression, aldehyde dehydrogenase 1 (ALDH1), stem cell-associated genes and proteins, and stem-cell associated histone modifier genes [23–26].

**Figure 1:**
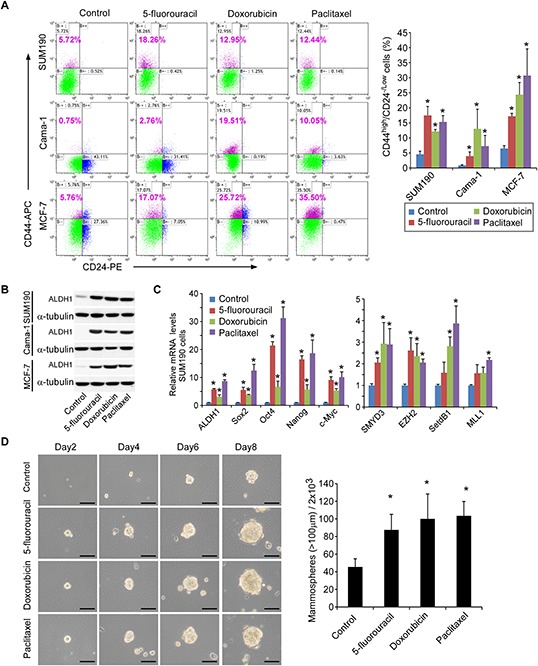
Breast CSCs are enriched after treatment with chemotherapeutic drugs **A.** SUM190, Cama-1 and MCF-7 breast cancer cells were cultured in the media as described previously [16] and treated with vehicle control, 5-fluorouracil (0.15 mM for SUM190 and MCF-7, 0.25 mM for Cama-1), doxorubicin (0.6 μM), and paclitaxel (15 nM) for 4d and recovered for 2d in the absence of drugs. The percentage of CD44^high^/CD24^−/low^ cells was assessed by flow cytometry. The left panel shows a representative experiment, and data in the right panel are means ± SD, *n* = 3; *p < 0.05. **B.** Western blot analysis of aldehyde dehydrogenase 1 (ALDH1) in SUM190, Cama-1, and MCF-7 cells treated with drugs as described above. α-tubulin was used as an internal loading control. **C.** Quantitative real-time PCR analysis of expression of stem cell-associated signature genes (*ALDH1*, *SOX2*, *OCT4*, *NANOG*, and *c-MYC*) and stem cell-associated chromatin modifiers (*SMYD3*, *EZH2*, *SETDB1*, and *MLL1*) in SUM190 cells after treatment with vehicle control, 5-fluorouracil, doxorubicin, and paclitaxel for 4 days and recovered for 2d in the absence of drugs. Data are relative amounts of mRNA compared to a reference gene *GAPDH* and represent means ± SD, *n* = 3; *p < 0.05. **D.** Representative images of the growing colonies at 2d, 4d, 6d, and 8d after treatment with drugs (phase-contrast). Scale bars = 100 μm. SUM190 cells were treated with vehicle control and drugs (5-fluorouracil, doxorubicin, or paclitaxel) for 4d and recovered in the absence of drugs for 2d. The dissociated single cells were then reseeded in ultra-low attachment plate (2 × 10^3^/well) to facilitate mammosphere formation in a mammosphere-culture medium. Scale bar = 100 μm. Data in right panel represent means ± SD, *n* = 3; *p < 0.05.

In comparison with the controls, exposure of SUM-190 cells to three different drugs resulted in a 2–3 fold increase in the CD44^high^/CD24^−/low^ subpopulation (Figure 1A, flow cytometry). A significant up-regulation of ALDH1 protein expression was also observed by Western blot (Figure 1B). To exclude cell type-dependence, we examined two additional breast cancer cell lines phenotypically different from SUM190 (inflammatory breast cancer cell line, invasive ductal carcinoma, ER-PR-HER2−/+), including MCF-7 (invasive ductal carcinoma, ER+PR+) and Cama-1 (adenocarcinoma, ER+PR−, oncogenic mutations in PTEN and p53, in-frame mutation in E-cadherin gene) [27]. As shown in Figure 1A and 1B, significant increases in the CD44^high^/CD24^−/low^ subpopulation and ALDH1 expression were also observed in these cell lines after treatment with three individual drugs, indicating that the phenomenon is not dependent on the type of tumor. Since CD44^high^/CD24^−/low^ and/or increases in ALDH1 have been widely used in characterization of breast CSCs [23–26], our results suggest that these chemotherapeutic drugs enrich the breast CSC subpopulation.

To further characterize CSCs after drug treatment, we assessed gene expression profiles. It has been shown that introduction of *OCT4*, *SOX2*, *KLF4* and *c-MYC* genes transformed non-tumorigenic MCF-10A mammary epithelial cells into tumorigenic CD44+/CD24^low^ cells with CSC properties [28], indicative of the crucial roles of these transcriptional factors in CSC development. Furthermore, over-expression of *SOX2*, *OCT4* or *NANOG* alone is sufficient to enhance tumorigenesis in a mouse model [29–32]; up-regulation of *c-MYC* has been reported to increase the CSC fraction by 150-fold, enabling tumor formation and serial propagation with as few as 500 cells [33]. We therefore performed Q-PCR and found that drug treatment upregulated the expression of these cancer stem cell-associated genes in all three cell lines, including *ALDH1*, *SOX2*, *c-MYC*, *OCT4*, and *NANOG* (Figure 1C and Supplementary Figure S2).

In addition to stem cell-associated genes, we also found that the expression of stem cell-associated histone modifier genes *EZH2*, *SETDB1*, *SMYD3*, and *MLL1* was elevated in drug-treated cells (Figure 1C and Supplementary Figure S2). It is well known that disruption of the histone modification patterns is one of the most common features of human tumors [34]. For example, polycomb protein EZH2 is essential in stem cell self-renewal and has been shown to promote expansion of breast CSCs [35], histone methyltransferase SETDB1 is a bona fide oncogene and contributes to tumorigenesis [34], histone lysine methyltransferase SMYD3 plays a pivotal role in the regulation of oncogenic signaling [36], and MLL creates epigenetic changes and maintains tumor propagating cells in response to Wnt/β-catenin signaling [37].

Finally, as a functional measure of CSCs, we performed mammosphere assays based on the ability of breast CSCs to generate multicellular spheroids in suspension culture [24, 38]. SUM190 cells were treated with 5-fluorouracil, doxorubicin, or paclitaxel for 4d and recovered for 2d in the absence of drugs. The drug-treated cells were then reseeded in an ultra-low 6-well plate (2000 cells/well) in the mammosphere-forming medium without further drug treatment. After 8 days, SUM190 cells pre-treated with drugs before reseeding formed approximately 2-fold more mammospheres than those pre-treated with vehicle (Figure 1D). Moreover, the drug-treated tumor cells grew faster and generated larger mammospheres than the vehicle-treated counterparts. Collectively, these data indicate that exposure to chemotherapeutic drugs leads to enrichment of CSCs in different types of breast cancer cells. This provides a basis for CSC-targeting therapy concurrent with or following chemotherapy.

### Cardamonin diminishes drug-selected CSCs

**Figure 2:**
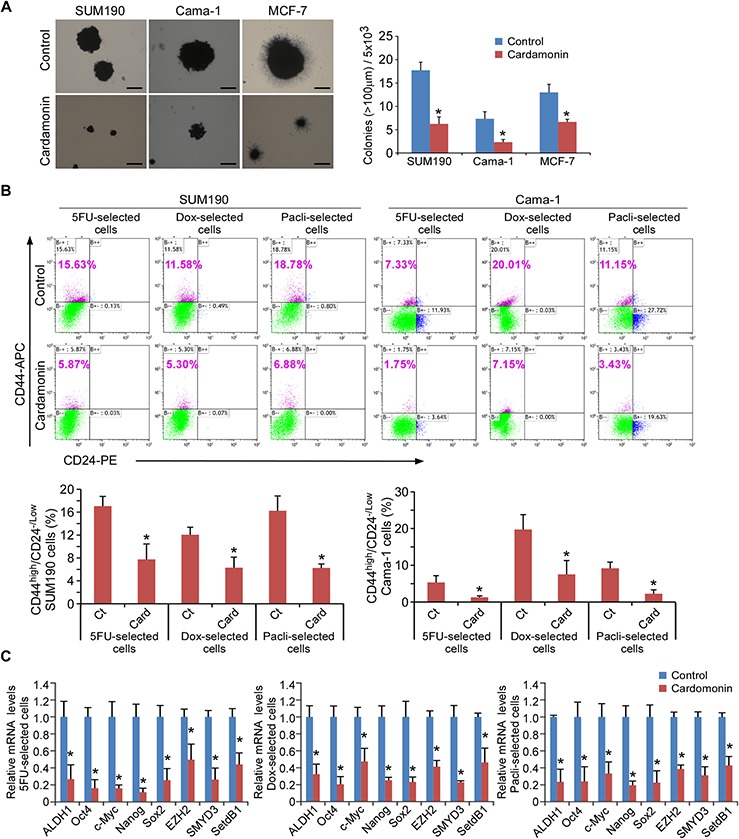
Cardamonin abrogates drug-selected CSCs **A.** Soft-agar colony formation assays. Cells were incubated in the presence or absence of 7.5 μM cardamonin for the duration of the assay. At day 17, colonies (>100 μm in diameter) were counted, and cell viability in colonies was determined by MTT. All experiments were performed in triplicate. Data in right panel represent means ± SD from three independent experiments. Scale bar = 100 μm. *p < 0.05. **B.** Percentage of CD44^high^/CD24^−/low^ cells assessed by flow cytometry. SUM190 and Cama-1 breast cancer cells in monolayer culture were treated with 5-fluorouracil (5FU-selected, 0.15 mM for SUM190, 0.25 mM for Cama-1), doxorubicin (Dox-selected, 0.6 μM), or paclitaxel (Pacli-selected,15 nM) respectively for 4d, followed by a 2d culture in the absence of chemotherapeutic drugs and in the absence (Ct) or presence of cardamonin (Card, 7.5 μM). Data represent means ± SD, *n* = 3; *p < 0.05. **C.** Q-PCR analysis of expression from stem cell-associated genes (*ALDH1*, *OCT4*, *c-MYC*, *NANOG* and *SOX2*) and stem cell-associated chromatin modifier genes (*EZH2*, *SMYD3* and *SETDB1*) in drug-selected SUM190 cells in the absence or presence of 7.5 μM cardamonin for 2d. Data are relative amounts of mRNA compared to a reference gene *GAPDH* and represent means ± SD, *n* = 3; *p < 0.05.

Our recent data showed that cardamonin inhibited the expression of key CSC-marker genes induced by inflammation [16]. We then asked whether cardamonin could inhibit *in vitro* growth and tumorigenic potential of breast CSCs that had survived after chemotherapeutic drug treatments. We first performed soft agar colony-forming assays, a method commonly used for examination of potential therapeutic agents in oncology [25, 39]. Notably, cardamonin resulted in a 2 to 3- fold decrease in colony-forming capacity in all three cell lines tested (Figure 2A). Since the ability to form mammosphere is a prominent feature of CSCs [40], these results suggest that cardamonin abrogates breast CSC function.

In the light of the above observations, we further investigated the potential role of cardamonin in the drug-selected breast cancer cells. After 4 days drug treatments, remaining drug-tolerant cancer cells were further treated with cardamonin or vehicle, and analyzed by flow cytometry. Consistently, cardamonin treatment decreased the CD44^high/^CD24^−/low^ subpopulation by 2 to 3-fold in drug-tolerant SUM190 cells and 3 to 4-fold in drug-tolerant Cama-1 cells (Figure 2B). Cardamonin also down-regulated the expression of stem-cell associated genes *ALDH1*, *SOX2*, *c-MYC*, *OCT4*, and *NANOG* and stem cell-associated histone modifier genes *EZH2*, *SETDB1*, and *SMYD3* in these drug-tolerant cells as determined by Q-PCR (Figure 2C and Supplementary Figure S3). Collectively, the results from soft agarose functional assays, flow cytometry, and “stemness”-associated gene expression analysis demonstrate that cardamonin abolishes CSCs that have been enriched by chemotherapeutic drugs. These results suggest that cardamonin could be used to target breast CSCs after completion of chemotherapy.

To understand cellular mechanisms underlying the action of cardamonin, we asked whether cardamonin could inhibit CSC enrichment by diminishing CSC properties or by causing CSC death in sorted CD44^high^/CD24^−/low^ CSCs. We fractionated CD44^high^/CD24^−/low^ CSC subpopulations from SUM190 and MDA-MB-231 (ER^−^PR^−^HER2^−^) breast cancer cell lines using fluorescence activated cell sorting (Supplementary Figure S4). We then seeded CSC subpopulations in ultra-low attachment plates and cultured them in mammosphere formation media in the presence or absence of cardamonin. This assay reflects the presence of self-renewing, gland-reconstituting stem cells within the population [25]. Notably, fractionated CSCs treated with cardamonin formed smaller and fewer mammospheres than those treated with the vehicle alone (Supplementary Figure S4A), suggesting a reduced self-renewal capacity. We also assessed the percentage of CSCs and non-CSCs in the fractionated CD44^high^/CD24^−/low^ subpopulation after vehicle or cardamonin treatment. While cardamonin treatment led to a 2-fold decrease in the CSC subpopulation compared to the vehicle control, it caused insignificant CSC death according to 7AAD staining in comparison to the vehicle control (Supplementary Figure S4B). Fractionated CD44^high^/CD24^−/low^ CSCs from both SUM190 and MDA-MB231 cell lines showed similar results (Supplementary Figure S4C). Additionally, increases in gene expression levels associated with CSCs (*ALDH1*, *OCT4*, *NANOG*, and *SOX2*) in CD44^high^/CD24^−/low^ subpopulations fractionated from both SUM190 and MDA-MB231 cells were lower after treatment with cardamonin (Supplementary Figure S4D). Taken together, these results suggest that cardamonin reduces CSC subpopulation through diminishing CSC properties instead of enhancing CSC death, implicating that cardamonin facilitates the conversion of breast CSCs to non-CSCs.

### Prevention of CSC enrichment when concurrent use of chemotherapeutic drugs and cardamonin

**Figure 3:**
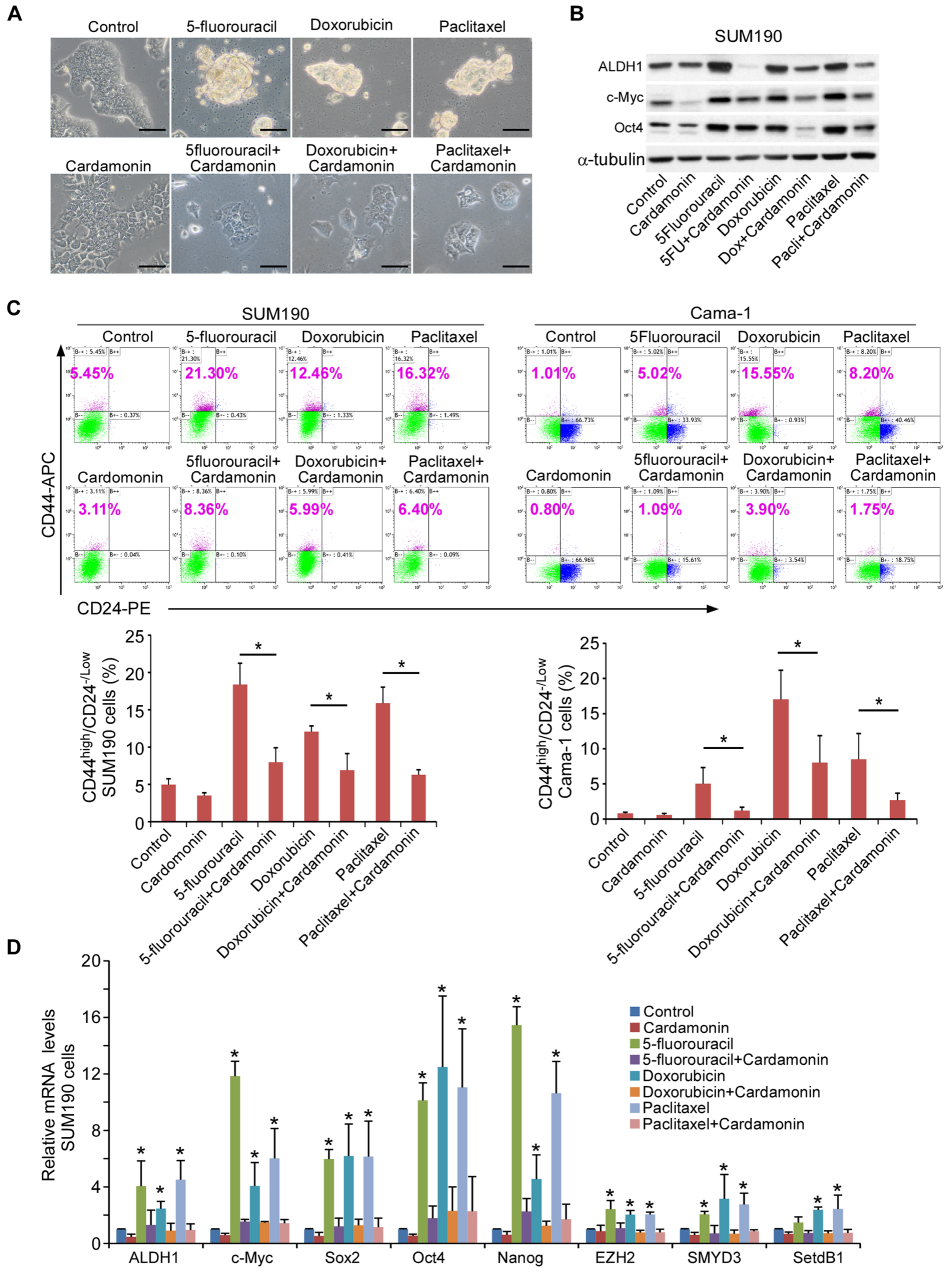
Co-treatment with cardamonin prevents the enrichment of CSCs induced by chemotherapeutic drugs **A.** Cardamonin abrogates the formation of spherical clusters induced by chemotherapeutic drugs. Representative images of SUM190 cells after treatment with 5-fluorouracil (0.15 mM), Doxorubicin (0.6 μM), or paclitaxel (15 nM) in the presence or absence of cardamonin (7.5 μM) for 4d. Scale bars = 100 μm. **B.** Western blot analysis of ALDH1, c-MYC, and SOX2 proteins for the indicated groups as shown in A. α-tubulin was used as an internal loading control. **C.** Flow cytometry analysis of CD44^high^/CD24^−/low^ subpopulation in SUM190 and Cama-1 cells after treatment with 5-fluorouracil (0.15 mM for SUM190, 0.25 mM for Cama-1), doxorubicin (0.6 μM), or paclitaxel (15 nM) in the presence or absence of cardamonin (7.5 μM) for 4d. Data represent means ± SD, *n* = 3; *p < 0.05. **D.** Q-PCR analysis of expression from stem cell-associated genes (*ALDH1*, *c-MYC*, *SOX2*, *OCT4* and *NANOG*) and stem cell-associated chromatin modifier genes (*EZH2*, *SMYD3* and *SETDB1*). SUM190 cells were treated as described in A. Results show relative amounts of mRNA compared to a housekeeping gene *GAPDH*. Data represent means ± SD, *n* = 3; *p < 0.05.

Since cardamonin abolished chemotherapeutic drug-selected CSCs (Figure 2), we asked whether cardamonin could also prevent CSC enrichment when used concurrently with chemotherapeutic drugs. We treated SUM190 cells with 5-fluorouracil, doxorubicin, or paclitaxel in the simultaneous absence or presence of cardamonin. In contrast to the cells treated with vehicle and grew as an adherent monolayer, SUM190 cells treated with individual drugs alone formed adherent mammosphere-like structures, which were effectively inhibited by cardamonin (Figure 3A). Western blot analysis showed that cells co-treated with cardamonin and chemotherapeutic drugs significantly diminished the up-regulation of ALDH1, c-MYC, and OCT4 proteins associated with CSC phenotypes (Figure 3B).

We further analyzed CD44^high^/CD24^−/low^ CSC subpopulation using flow cytometry. Cardamonin almost completely abolished the up-regulated CD44^high^/CD24^−/low^ subpopulation induced by concurrent addition of three individual drugs in SUM190 and Cama-1 cells (Figure 3C). We also analyzed gene expression profiling using Q-PCR. In comparison to the cells treated with chemotherapeutic drugs alone, cardamonin inhibited the expression of stem cell-associated genes *ALDH1*, *SOX2*, *c-MYC*, *OCT4*, and *NANOG* as well as stem cell-associated histone modifier genes *EZH2*, *SETDB1*, and *SMYD3* (Figure 3D), suggesting that cardamonin sufficiently prevents the enrichment of CSCs when simultaneously used with chemotherapeutic drugs. Additionally, emerging evidence has shown that histone modification results in tumor cell plasticity and dynamic phenotypic heterogeneity, which is an important determinant of the effectiveness of chemotherapy [41, 42]. It is possible that repression of some histone modifier genes will facilitate chemotherapeutic efficacy.

### Cardamonin inhibits chemotherapeutic drug-induced inflammatory cytokines and NF-κB and Stat3 activation in breast cancer cells

**Figure 4:**
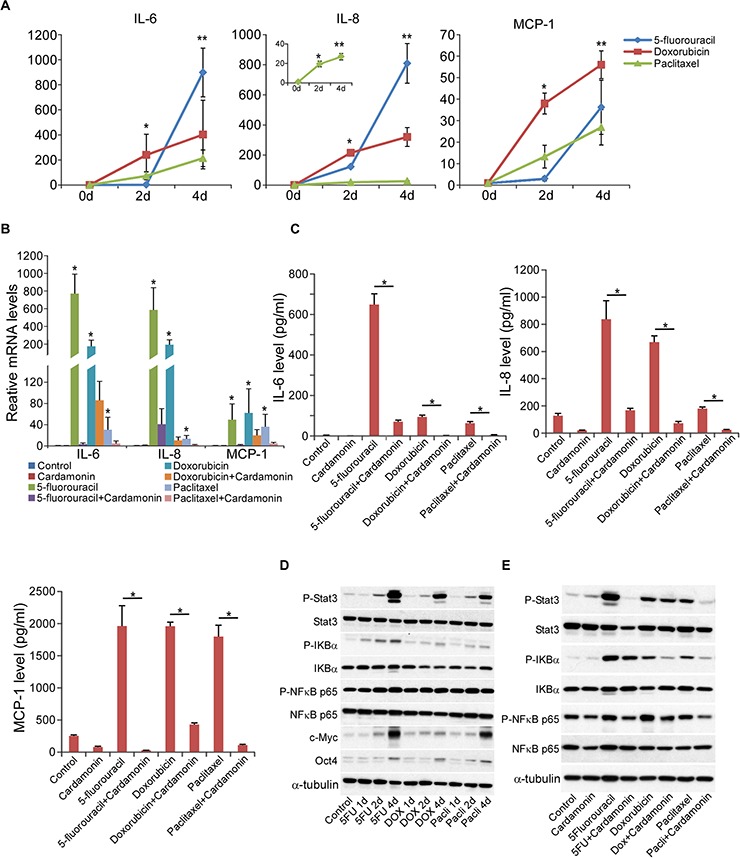
Cardamonin inhibits chemotherapeutic drug-induced inflammatory signature and NF-κB and Stat3 activation **A.** Changes of *IL-6*, *IL-8*, and *MCP-1* gene expression (Q-PCR analysis) at different time points (0d, 2d, and 4d) in SUM190 cells after treatment with 5-fluorouracil (0.15 mM), Doxorubicin (0.6 μM), or paclitaxel (15 nM). *GAPDH* mRNA was used to normalize relative levels of cytokine mRNA. Data represent means ± SD, *n* = 4; *p < 0.05. **B.** *IL6*, *IL-8*, and *MCP-1* gene expression (Q-PCR analysis) after treatment with 5-fluorouracil (0.15 mM), Doxorubicin (0.6 μM), or paclitaxel (15 nM) in the presence or absence of cardamonin (7.5 μM) for 4d. *GAPDH* mRNA was used to normalize relative levels of cytokine mRNA. Data represent the average ± SD, *n* = 4; *p < 0.05. **C.** Concentrations of IL6, IL-8, and MCP-1 proteins in the supernatants after treatment with 5-fluorouracil (0.15 mM), Doxorubicin (0.6 μM), or paclitaxel (15 nM) in the presence or absence of cardamonin (7.5 μM) for 4d. Cytokine levels were determined using a fluorescence-based multiplex cytokine assay kit (Eve Technologies). Data represent means ± SD, *n* = 3; *p < 0.05. **D.** Western blot analysis of CSC-associated markers c-MYC and OCT4, phosphorylation of NF-κB, IκBα, and STAT3 in SUM190 cells at different time points (0d, 1d, 2d, and 4d) after treatment with chemotherapeutic drugs (5-fluorouracil, Doxorubicin, or paclitaxel). α-tubulin was used as an internal loading control. Data are all from one experiment, and three experiments were performed with similar results. **E.** Cardamonin diminishes chemotherapeutic drug-induced activation of NF-κB, IκBα, and STAT3 (Western blot analysis). Data are all from one experiment, and three experiments were performed with similar results.

Inflammatory cytokines, NF-κB and Stat3 activation have been closely associated with cancer progression and CSC development [16, 17, 43–46]. We therefore determined whether chemotherapy drugs that led to CSC enrichment (Figure 1) would enhance the expression of inflammatory cytokines in breast cancer cells. Indeed, gene expression levels of *IL-6*, *IL-8*, and human monocyte chemoattractant protein-1 (*MCP-1*) were robustly elevated and also correlated positively with progressively increased CSC-marker genes in a time-dependent manner after treatment with doxorubicin, 5-fluorouracil, and paclitaxel (Figure 4A and 1C). Strikingly, cardamonin almost completely abrogated chemotherapeutic drug-upregulated transcription of *IL-6*, *IL-8*, and *MCP-1* (Figure 4B), and also diminished protein expression of IL-6, IL-8, and MCP-1 (Figure 4C). These results indicate that cardamonin is capable of repressing key inflammatory cytokines closely associated with the development of CSCs.

In addition, chemotherapeutic drug-treatment augmented the activation (phosphorylation) of nuclear factor-kappa B (NF-κB), nuclear factor of kappa light polypeptide gene enhancer in B-cells inhibitor alpha (IκBα) and signal transducer and activator of transcription 3 (Stat3) in a time-dependent manner, which was also positively correlated with the increased CSC-marker proteins c-MYC and OCT4 (Figure 4D). This observation is partially consistent with a report showing that cardamonin inhibited NF-κB and IκB in macrophage cells [47]. It is well known that activation of NF-κB and Stat3 inflammatory pathways will upregulate the secretion of various cytokines including IL-6, IL-8 and MCP-1 and promote the development of CSCs [16, 17, 43, 46]. Significantly, cardamonin abrogated chemotherapeutic drug-induced activation of NF-κB and STAT3 in SUM190 and MCF-7 cells (Figure 4E and Supplementary Figure S5). Together, these results suggest a possible molecular mechanism underlying inhibitory effects of cardamonin on CSC enrichment induced by chemotherapeutic drugs.

### Cardamonin enhances chemotherapeutic drug efficacy in retardation of tumor growth while reducing CSC subpopulation *in vivo*

**Figure 5:**
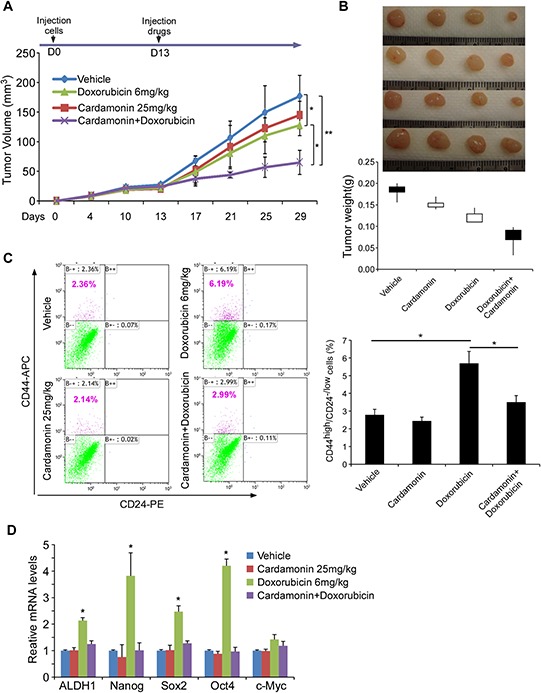
Cardamonin enhances doxorubicin efficacy in retardation of tumor growth while reducing doxorubicin-enriched CSC subpopulation *in vivo* **A–B.** SUM190 breast cancer cells were implanted into the mammary fat pads of athymic nude mice. When the tumor reached a mean diameter of 4 mm (day 13), mice were randomized into 4 groups and intraperitoneally injected with vehicle, doxorubicin alone (6 mg/kg on days 13, 18, and 23), cardamonin alone (25 mg/kg on every other day starting from day13), or doxorubicin + cardamonin. Tumor volumes were determined every 3–4 d (A) Tumors were harvested and tumor weights were measured on day 29 (B) Data represent means ± SD, *n* = 4–6; *p < 0.05, **p < 0.001. **C.** Percentage of CD44^high^/CD24^−/low^ cells in tumors determined by flow cytometry. Doxorubicin injection retards tumor growth but also significantly increases CSC pool. In contrast, co-injection with cardamonin abrogates doxorubicin-enriched CSCs. Data represent means ± SD, *n* = 4; *p < 0.05. **D.** Gene expressions of tumor samples determined by Q-PCR. CSC-associated markers are up-regulated in tumor samples from mice injected with doxorubicin alone but abolished when co-administration with cardamonin. Data represent means ± SD, *n* = 3; *p < 0.05.

To determine whether cardamonin exhibits similar effects *in vivo*, we transplanted SUM190 cells into the mammary fat pads of athymic nude mice. When the tumor reached a mean diameter of 4 mm after 13 days of implantation, mice were randomized into 4 groups and injected intraperitoneally with vehicle, doxorubicin (6 mg/kg on days 13, 18, and 23), cardamonin (25 mg/kg once every other day from days 13) or a combination of both. As shown in Figure 5A and 5B, treatment with doxorubicin alone impeded tumor growth (*p* < 0.05) while treatment with cardamonin alone only mildly hindered tumor growth in comparison to control group (*p* > 0.05). Significantly, the combination of doxorubicin and cardamonin markedly reduced tumor burden in comparison to each of the other three groups (*p* < 0.05 - *p* < 0.001, Figure 5A and 5B), suggesting that cardamonin enhances chemotherapy efficacy.

To determine whether cardamonin with doxorubicin reduces the CSC pool *in vivo*, we harvested tumors after three cycles of treatment and assessed the CD44^high^/CD24^−/low^ subpopulation using flow cytometry. Notably, the CSC population in tumors harvested from mice receiving doxorubicin alone increased 2.5-fold over those receiving vehicle alone, although the tumor weights were lower and sizes smaller (Figure 5C). In contrast, co-administration of cardamonin and doxorubicin not only remarkably reduced the tumor growth but also abolished doxorubicin-enriched CSCs in mouse xenografts (Figure 5C).

We further assessed the expression levels of several “stemness” genes of xenografts. Tumors from doxorubicin-treated mice showed higher levels of *ALDH1*, *SOX2*, *OCT4* and *NANOG* expression compared to vehicle controls (Figure 5D). In contrast, co-administration of cardamonin and doxorubicin almost completely abolished doxorubicin-upregulated “stemness” genes (Figure 5D). Thus, the key therapeutic advantage of cardamonin in combination with conventional chemotherapy may be the effective elimination of both the bulk of the tumor and tumorigenic CSCs.

## DISCUSSION

Chemotherapeutic drug resistance represents one of the major unmet clinical challenges in breast cancer treatment. Recent studies have suggested that eradication of breast CSCs that are resistant to chemotherapy is required to achieve a complete remission and prevent disease recurrence [7–9, 13, 41, 48]. In this study, we demonstrate that different breast cancer cell lines after treatment with clinically relevant concentrations of 5-fluorouracil, doxorubicin, or paclitaxel enrich CSCs, increase expression of IL-6, IL-8, and MCP-1 cytokines, and up-regulate activation of NF-κB and Stat3 pathways.

IL-6 and IL-8 play an essential role in breast tumorigenesis, associate with poor patient survival, and are implicated in the maintenance of breast CSCs and chemotherapy resistance, particularly in basal subtype and triple negative breast cancer [5, 49–51]. MCP-1 has been found to enhance stem cell phenotypes and CSC self-renewal in breast cancer [46], and facilitate the generation of induced pluripotent stem cells [52]. It is also well known that NF-κB and Stat3 pathways govern cytokine productions in response to different stimuli, are associated with drug resistance, and regulate tumor angiogenesis and invasiveness [16, 44, 45, 50, 53–55]. Significantly, our results show that cardamonin is capable of effectively repressing IL-6, IL-8 and MCP-1 up-regulation and NF-κB and Stat3 activation, suggesting a possible mechanism underlying cardamonin inhibitory effects on CSC enrichment induced by chemotherapeutic drugs.

Interestingly, our results also show that cardamonin suppresses stem cell-associated histone modifier genes *EZH2*, *SETDB1*, *SMYD3*, and *MLL1* induced by chemotherapeutic drugs (Figure 3D). Histone modification plays an important role in tumor cell plasticity and phenotypic heterogeneity [34–37, 56]. Our observation that cardamonin facilitates the conversion of breast CSCs to non-CSCs while insignificantly enhancing CSC death may associate with its inhibitory effect on histone modifiers. Particularly, EZH2 physically links β-catenin on the target gene promoters of cyclin B1 and c-Myc [57]. EZH2 has also been shown to promote expansion of breast CSCs through activation of Erk-β-catenin signaling [35]. Additionally, EZH2 activates the transcription of NF-κB target genes by forming a ternary complex with two NF-κB subunits in triple negative MDA-MB-231 cells [58]. As such, although it remains unknown whether and to what extent inhibition of this histone modifier by cardamonin is through repressing NF-kB and Stat3 signaling, it is possible that crosstalk between EZH2 (or SETDB1, SMYD3, MLL1) and NF-κB and Stat3 might be an important determinant in drug resistance in breast cancer, warranting further investigation.

In conclusion, cardamonin suppresses existing breast CSCs after chemotherapeutic drug treatments, prevents the enrichment of new CSCs during chemotherapeutic drug treatments, and enhances the efficacy of chemotherapeutic drugs by reducing tumor burden as well as the CSC pool *in vivo*. Cardamonin, which is found in many plant species [15], seems to be a promising candidate in CSC-targeted therapies either concurrently used with chemotherapeutic drugs or after chemotherapeutic treatment.

## MATERIALS AND METHODS

### Cell culture and reagents

Breast cancer cell line SUM190 were obtained from Asterand (Detroit, MI, USA) and cultured in Hams F-12 media (Mediatech, Manassas, VA, USA) containing 5 μg/ml insulin, 1 μg/ml hydrocortisone, antibiotics (penicillin/streptomycin), and 2% of fetal bovine serum (HyClone, Logan, UT, USA). Medium for SUM190 cell culture was further supplemented with 5 mM ethanolamine, 10 mM HEPES, 5 μg/ml transferrin, 6.6 ng/ml 3,3′,5-triiodo-L-thyronine sodium salt, 8.7 ng/ml sodium selenite, and 1 mg/ml bovine serum albumin. Cells were cultured at 37°C in a 5% CO2 incubator. Breast cancer cell lines MCF-7, MDA-AB-231 and Cama-1 were purchased from the American Type Culture Collection (Manassas, VA) and maintained in DMEM-F12 (1:1) supplemented with 10% Fetal Bovine Serum (FBS). Doxorubicin hydrochloride, 5-fluorouracil, paclitaxel, insulin, hydrocortisone, HEPES, and bovine serum albumin (BSA) were purchased from Sigma-Aldrich (St. Louis, MO, USA). Cardamonin was purchased from TOCRIS bioscience (Ellisville, MO, USA). B27 supplement was purchased from Fisher (Cat. 17504-044), and EGF and bFGF from RD (Cat. 236-EG-200 and 234-FSE).

### Xenograft tumor growth

All mouse experimentation was conducted in accordance with standard operating procedures approved by the Animal Care Committee at the University of Ottawa. Athymic nude mice (6–8 week old, female, 20 to 25 g body weight) were obtained from Charles River Laboratories. To establish breast cancer xenografts in nude mice, SUM190 cells were mixed with Matrigel and injected under aseptic conditions into mammary fat pads of nude mice (*n* = 4–8 for each group, 2 × 10^6^ cells per fat pad). The tumor was monitored and evaluated every 2–3 days with calipers. Tumors were measured in 2 dimensions, and volume was calculated according to the formula: *V* = 0.5 ((length) × (width)^2^. When the tumor reached a mean diameter of 4 mm (approximately day 13), mice were randomized into 4 groups: vehicle control, doxorubicin (6 mg/kg on days 13, 18, and 23), cardamonin (25 mg/kg on every other day from days 13) or doxorubicin and cardamonin. At the end of drug treatment, mice were humanely euthanized and tumors were harvested and measured (weight and volume). Xenografted tumors were further analyzed by flow cytometry and Q-PCR.

### Flow cytometry analysis

Cancer cells dissociated from transplanted tumor tissues or from culture plates were counted and re-suspended in 100 μl of HBSS containing 2% heat-inactivated fetal bovine serum (HIFS) and 10^5^ cells. Five microliters of mouse IgG solution (1 mg/ml) was added and incubated on ice for 10 min. According to the manufacturer's recommendation, appropriate antibodies were added and incubated for 30 min on ice. The cells were then washed twice with HIFS and re-suspended in 0.2 ml of HIFS that contained 7-aminoactinomycin D (7AAD, 1 μg/ml, final concentration) to exclude dead cells. Appropriate fluorochrome-conjugated isotype matched antibodies were used as negative controls. Antibodies used were anti-CD44 (APC), anti-CD24 (PE), which were purchased from BD Pharmingen. Flow cytometry was performed on a Cyan-ADP 9 and data analyzed with Kaluza software (Beckman Coulter, USA).

### Fractionation of CSC and non-CSC subpopulations from breast cancer cells

To separate CSCs from non-CSCs (SUM190 and MDA-MB-231 cell lines), single-cell suspensions were stained with CD44 antibody (APC-conjugated) and CD24 antibody (PE-conjugated) for 30 min, analyzed and sorted by MoFlo Astrios - Sorter (Beckman Coulter). Isolation gates, including histogram markers and dot plot quadrants, were set based on respective IgG isotype controls. Purity was determined immediately after sorting and was approximately >90%. CSCs are defined by CD44^high^/CD24^−/low^ subpopulation, whereas non-CSCs are defined by CD44^−/low^/CD24^high^ subpopulation. CSCs were reseeded on ultralow-attachment plates and cultured in DMEM/F12 medium containing B27 supplement, 20 ng/ml of EGF, and 20 ng/ml of basic FGF. Plates were incubated at 37°C in 5% CO_2_ to allow mammosphere formation in the presence or absence of cardamonin followed by mammosphere formation assays, qPCR and flow cytometry analyses.

### Mammosphere formation assays

SUM190 cells were treated with vehicle control, 5-fluorouracil, doxorubicin, or paclitaxel for 4d, and then allowed to recover in the absence of treatment for 2 days. The cells (2 × 10^3^/well) were reseeded in ultralow attachment plate in sphere medium (DMEM/F12 medium containing B27 supplement, 20 ng/ml of EGF and 20 ng/ml of basic FGF). The plates were incubated at 37°C in 5% CO2 for 8 days to allow mammosphere formation. Colonies (>100 μm in diameter) of each groups were counted.

### Soft agar colony formation

A soft-agar assay was performed on 12-well plates with a base layer of 0.5% agarose gel containing DMEM. To generate the cell layer, 5 × 10^3^ cells/well were suspended in 0.35% top agarose gel in DMEM/F12 medium containing B27 supplement, 20 ng/ml of EGF and 20 ng/ml of basic FGF. SUM190, MCF-7, and Cama-1 cells were cultured in the presence or absence of cardamonin, incubated at 37°C in 5% CO2 for 17 days to allow colony formation. Cell viability was determined by staining with 3-(4,5-dimethylthiazol-2-yl)-2,5-diphenyl tetrazolium bromide (MTT, 1 mg/ml). Colonies (>100 μm in diameter) of each cell line were counted. All experiments were performed in triplicate, and data are presented as means ± SD.

### Western blot analysis

For Western blot analysis, cells were harvested and lysates prepared using RIPA buffer (Sigma) and sub-cellular fractions prepared using the NE-PER Nuclear Protein Extraction Kit (Thermo Scientific) containing protease inhibitor cocktails (Roche, Mannheim, Germany). Protein concentration was determined using a Bio-Rad DC protein assay kit (Bio-Rad, Hercules, CA, USA). Subsequently, 25 μg–30 μg of total protein for each sample was loaded onto an 8–12% SDS-polyacrylamide gel for electrophoresis and then transferred to a PVDF membrane. Protein was identified by incubating the membrane with primary antibodies, followed by horseradish peroxidase-conjugated secondary antibodies and an enhanced chemiluminescence solution (Pierce, Thermo Scientific, USA). Antibodies used in this study include: anti-c-Myc polyclonal antibody (D84C12, Cat. 5605), anti-phospho-Stat3(Tyr705) monoclonal antibody (1:1000, D3A7, Cat. 9145), anti-Stat3 monoclonal antibody (1:1000,124H6, Cat. 9139) from Cell Signaling (Danvers, MA, USA); anti-ALDH1A1 antibody (1:1000, ab105920), anti-OCT4 antibody (1:1000, ab137427) from ABCAM (Cambridge, MA, USA); anti-α-tubulin monoclonal antibody (T9026) from Sigma-Aldrich (St. Louis, MO, USA); anti-NF-κB p65 monoclonal antibody (1:1000, 112A1021), anti-phospho-NF-κB p65 pSer 536 monoclonal antibody (1:1000, T.849.2), anti-I-kappa-B-alpha monoclonal antibody (1:1000, T.937.7), and anti-phospho-I-kappa-B-alpha pSer32/36 monoclonal antibody (1:1000, H.709.9) from Thermo scientific (Rockford, USA).

### Quantitative real-time PCR (Q-PCR)

Total RNAs were extracted using RNeasy kit (QIAGEN) and real-time Q-PCR analysis was performed using Bio-Rad MyiQ (Bio-Rad, USA) as previously described [59]. The conditions for Q-PCR reactions are: one cycle at 95°C for 20 seconds, followed by 40 cycles at 95°C for 3 seconds and annealing at 60°C for 30 seconds. Results were normalized to the housekeeping gene glyceraldehyde 3-phosphate dehydrogenase (GAPDH). Relative expression level of genes from different groups were calculated with the ^2ΔΔ^CT method and compared with the expression level of the corresponding gene in control cells. The primers used were listed in Table 1.

**Table 1:** Primers used in Q-PCR for measuring gene expression relative to GAPDH

Gene names	Forward	Reverse
IL6	AACAACCTGAACCTTCCAAAGA	TCAAACTCCAAAAGACCAGTGA
IL8	ATGACTTCCAAGCTGGCCGTGGCT	TCTCAGCCCTCTTCAAAAACTTCTC
MCP-1	AAGATCTCAGTGCAGAGGCTCG	TTGCTTGTCCAGGTGGTCCAT
Oct4	CTGCAGTGTGGGTTTCGGGCA	CTTGCTGCAGAAGTGGGTGGAGGAA
Nanog	CATGAGTGTGGATCCAGCTTG	CCTGAATAAGCAGATCCATGG
ALDH1	CGCAAGACAGGCTTTTCAG	TGTATAATAGTCGCCCCCTCTC
c-Myc	TTCTCTCCGTCCTCGGATTCTCTG	TCTTCTTGTTCCTCCTCAGAGTCG
Sox2	CATCACCCACAGCAAATGACAGC	TTGCGTGAGTGTGGATGGGATTG
EZH2	CAAGCAGTGCCCGTGCTA	AGCGGCTCCACAAGTAAGACA
SMYD3	CCCAACTGTTCGATTGTGTTCA	TCCTCTCCCACCTCGATGTC
MLL1	CGGGAAAAGTATTACGACAG	CACACGAGTGATTGATGAAG
SETDB1	GACTCTCTGAGACAACTTCCAAGGA	CAGGGATTGAGGGAGGAACA
GAPDH	ACAGTCAGCCGCATCTTCTT	GACAAGCTTCCCGTTCTCAG

### Half maximal inhibitory concentration of chemotherapeutic drugs

To titrate the half maximal inhibitory concentration of each chemotherapeutic drug, 2000 cells per well were seeded into 96-well plates for each cell line. One day after seeding, drugs were added in five replicates for each concentration. After 4d, MTT reagent (tetrazolium) was added for 4 h and the reaction was stopped by removing MTT and adding DMSO (150 μL) to each well to dissolve formazan crystals. Absorbance was measured at 570 nm.

### Human cytokine arrays

Cell-free supernatants from chemotherapeutic drug-stimulated SUM190 cells were collected after 4d and used for cytokine analysis. An 11-plex cytokine profiling kit was used (Eve Technologies, Calgary, Alberta, Canada), including IFN-γ, GM-CSF, interleukin 1β, interleukin 2, interleukin 4, interleukin 6, interleukin 8, interleukin10, interleukin 12, MCP-1, and tumor necrosis factor α.

### Statistical analyses

Data are expressed as mean ± Standard Deviation (SD) unless specified elsewhere. Statistical significance was determined using a Student's *t* test, or ANOVA wherever appropriate. Results were considered significant when *p* < 0.05, *p* < 0.001.

## SUPPLEMENTARY FIGURES


