# Supplementary material for: Cardamonin reduces chemotherapy-enriched breast cancer stem-like cells in vitro and in vivo
Source: Oncotarget. 2015 Oct 17;7(1):771–85. doi: 10.18632/oncotarget.5819 (PMC4808032; doi:10.18632/oncotarget.5819)
Supplement: Supplementary file 1 [file oncotarget-07-0771-s001.pdf]

SUPPLEMENTARY FIGURES

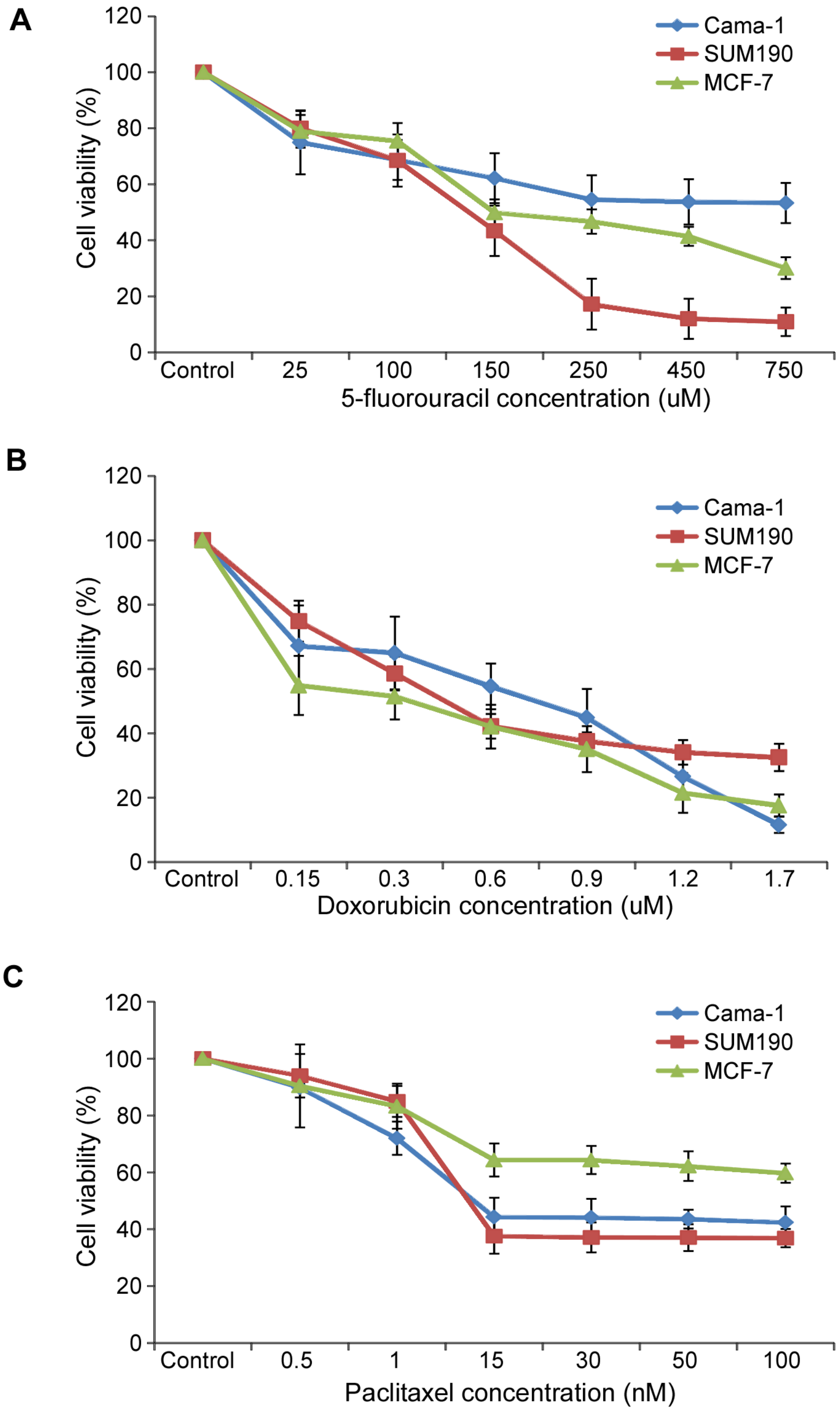

Supplementary Figure S1: Titration of half maximal inhibitory concentration of 5-fluorouracil, doxorubicin, and paclitaxel in SUM190, Cama-1, and MCF-7 cells. Cell viability was calculated by MTT assay at 4d after addition of various doses of 5-fluorouracil **A.** doxorubicin **B.** and paclitaxel **C.** Data represent means ± SD of three independent experiments.

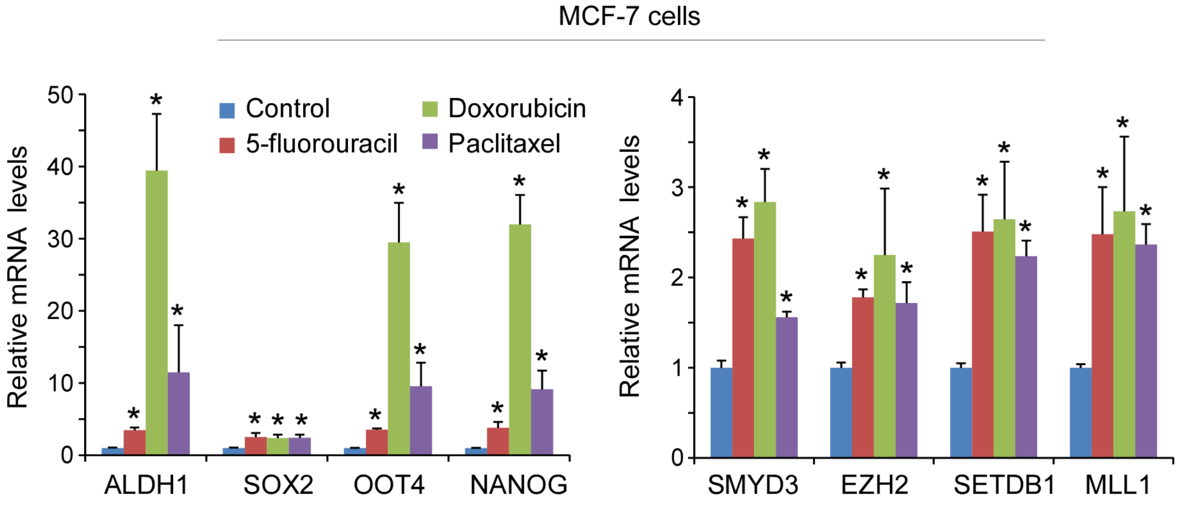

Supplementary Figure S2: Genes associated with cancer stem cells are up-regulated in MCF-7 breast cancer cells after treatment with chemotherapeutic drugs. Q-PCR analysis of stem cell-associated signature genes (*ALDH1, SOX2, OCT4, NANOG*, and *c-MYC*) and stem cell-associated chromatin modifiers (*SMYD3, EZH2, SETDB1*, and *MLL1*) in MCF-7 cells after treatment with vehicle control, 5-fluorouracil (0.15 mM), doxorubicin (0.6 μM), and paclitaxel (15 nM) for 4d, followed by 2d of recovery in the absence of any drugs. The conditions for Q-PCR reactions are: one cycle at 95°C for 20 seconds, followed by 40 cycles at 95°C for 3 seconds and annealing at 60°C for 30 seconds. All samples were normalized against GAPDH mRNA. Data represent means ± SD; $n = 3$; $*p < 0.05$.

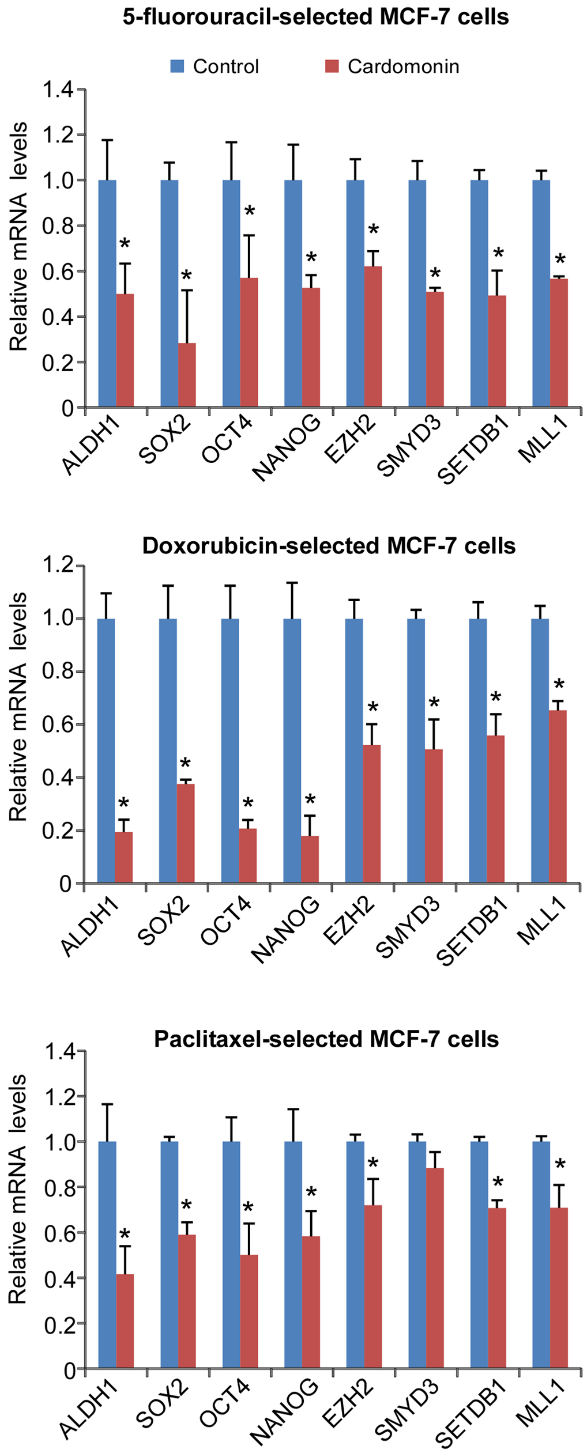

Supplementary Figure S3: Cardamonin suppresses CSC-associated gene expression induced by chemotherapeutic drugs in MCF-7 cells. MCF-7 breast cancer cells in monolayer culture were pre-treated with 0.15 mM 5-fluorouracil, 0.6 μM doxorubicin, or 15 nM paclitaxel for 4d, followed by a 2d culture in the absence of chemotherapeutic drugs and in the absence or presence of 7.5 μM cardamonin. Q-PCR analysis of expression of stem cell-associated genes (*ALDH1, SOX2, OCT4*, and *NANOG*) and stem cell-associated chromatin modifier genes (*SMYD3, EZH2*, and *SETDB1*). The conditions for Q-PCR reactions are the same as described in Supplementary Figure S1. Data are relative amounts of mRNA compared to a reference gene GAPDH and represent means ± SD; $n = 3$; $*p < 0.05$.

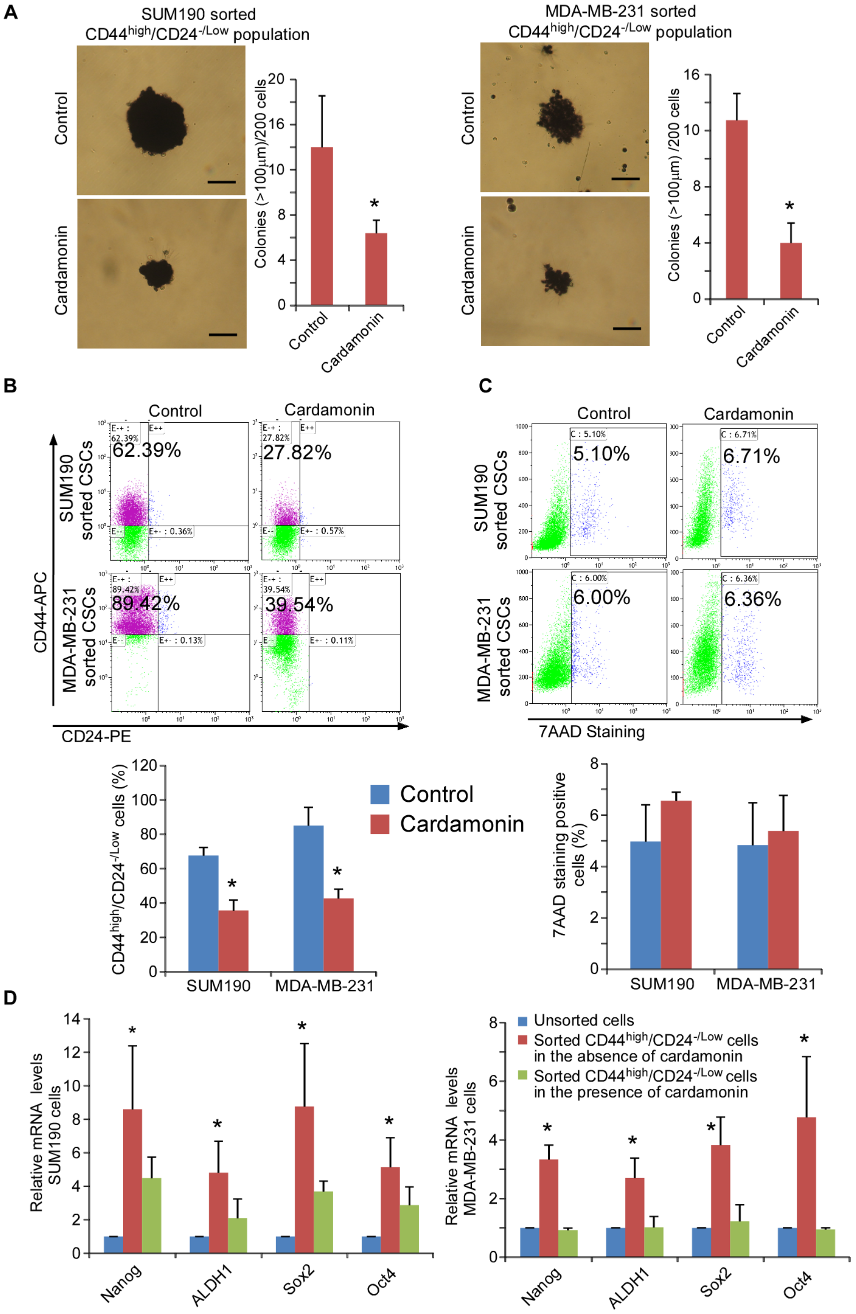

Supplementary Figure S4: Cardamonin inhibits CSC properties of sorted breast $CD44^{high}CD24^{-/low}$ cells and facilitates the conversion of CSCs to non-CSCs. A. Cardamonin inhibits mammosphere formation of sorted CSC subpopulation. The sorted $CD44^{high}CD24^{-/low}$ populations of SUM190 cells and MDA-MB-231 cells were re-seeded in mammosphere culture conditions and treated with vehicle (control) or cardamonin (7.5 µM) for 6d to assess their mammosphere-formation capacity. Cell viability in colonies was determined by MTT assays. Colonies > 100 µm in diameter were counted. All experiments were performed in triplicate. Data represent means ± SD. Scale bar = 100 µm. *p <0.05. **B.** Cardamonin facilitates the conversion of CSCs to non-CSC subpopulation. The percentages of sorted $CD44^{high}CD24^{-/low}$ SUM190 cells and MDA-MB-231 cells after treatment with vehicle or cardamonin (7.5 µM) for 4d were assessed by flow cytometry for the percentage of $CD44^{high}CD24^{-/low}$ and $CD44^{-/low}CD24^{high}$ subpopulation. Data represent means ± SD, $n = 3$; *$p < 0.05$. **C.** Cardamonin does not cause significant cell death of sorted breast CSCs. Cell viability of sorted $CD44^{high}CD24^{-/low}$ cells was assessed by 7-AAD staining followed by flow cytometric analysis. There were no significant differences in the percentage of dead cells after treatment with cardamonin compared to vehicle control in sorted SUM190 and MDA-MB-231 CSCs. Data represent means ± SD, $n = 3$; *$p < 0.05$. **D.** Q-PCR analyses of the expression of CSC-marker genes (*NANOG, ALDH1, OCT4*, and *SOX2*) in the unsorted, sorted $CD44^{high}CD24^{-/low}$ and sorted $CD44^{-/low}CD24^{high}$CSCs after treatment with cardamonin (7.5 µM) and vehicle in SUM190 and MDA-MB-231 cells. Data represent means ± SD, $n = 3$; *$p < 0.05$.

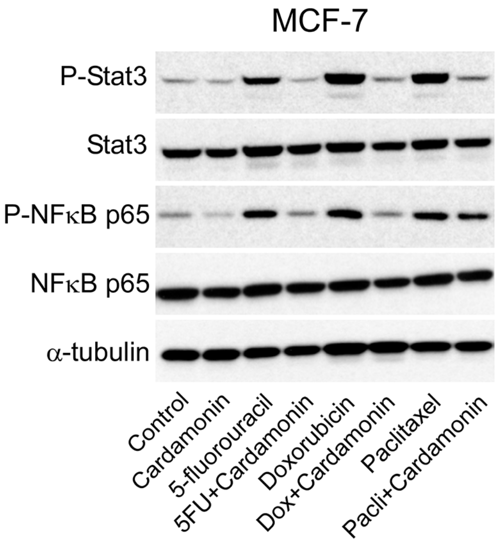

Supplementary Figure S5: Cardamonin inhibits chemotherapeutic drug-induced NF-kB and Stat3 activation in MCF-7 cells. Western blot analysis of phosphorylated (p)-NF-κB and p-Stat3 levels in MCF-7 cells after treatment with 5-fluorouracil (5-FU, 0.15 mM), Doxorubicin (Dox, 0.6 μM), or paclitaxel (Pacli, 15 nM) in the presence or absence of cardamonin (7.5 μM). α-tubulin was used as an internal loading control.
